# Long-Term Effectiveness and Safety of Tafluprost, Travoprost, and Latanoprost in Korean Patients with Primary Open-Angle Glaucoma or Normal-Tension Glaucoma: A Multicenter Retrospective Cohort Study (LOTUS Study)

**DOI:** 10.3390/jcm10122717

**Published:** 2021-06-19

**Authors:** Joon-Mo Kim, Kyung-Rim Sung, Hwang-Ki Kim, Sang-Woo Park, Eun-Ji Lee, Jin-Wook Jeoung, Hae-Young Lopilly Park, Jaehong Ahn, Chungkwon Yoo, Chan-Yun Kim

**Affiliations:** 1 Department of Ophthalmology, Kangbuk Samsung Hospital, Sungkyunkwan University School of Medicine, Seoul 03181, Korea; kjoonmo1@gmail.com; 2 Department of Ophthalmology, College of Medicine, University of Ulsan, Asan Medical Center, Seoul 05505, Korea; sungeye@gmail.com; 3 Department of Ophthalmology, Kim’s Eye Hospital, Myung-Gok Eye Research Institute, Konyang University, Seoul 07301, Korea; eye1001@kimeye.com; 4 Department of Ophthalmology and Research Institute of Medical Sciences, Chonnam National University Medical School and Hospital, Gwangju 61469, Korea; exo70@naver.com; 5 Department of Ophthalmology, Seoul National University College of Medicine, Seoul National University Bundang Hospital, Seongnam 13620, Korea; opticdisc@gmail.com; 6 Department of Ophthalmology, Seoul National University College of Medicine, Seoul National University Hospital, Seoul 03080, Korea; neuroprotect@gmail.com; 7 Department of Ophthalmology, Seoul St Mary’s Hospital, College of Medicine, The Catholic University of Korea, Seoul 06591, Korea; lopilly@catholic.ac.kr; 8 Department of Ophthalmology, Ajou University School of Medicine, Suwon 16499, Korea; chriseye68@gmail.com; 9 Department of Ophthalmology, Korea University College of Medicine, Seoul 02841, Korea; ckyoomd@korea.ac.kr; 10 Institute of Vision Research, Department of Ophthalmology, Severance Hospital, Yonsei University College of Medicine, Seoul 03722, Korea

**Keywords:** glaucoma, prostaglandin analogs, tafluprost, travoprost, latanoprost

## Abstract

This multicenter retrospective cohort study compared the effectiveness and safety of long-term tafluprost, travoprost, or latanoprost in patients with primary open-angle glaucoma (POAG) or normal-tension glaucoma (NTG). Data were extracted from electronic medical records of 300 patients treated with tafluprost, travoprost, or latanoprost for >6 months. Propensity matching for age and sex was used for effectiveness and safety comparisons. The primary endpoint was visual field (VF) progression via mean deviation (MD) slope. Secondary endpoints were change of MD, intraocular pressure, pattern standard deviation, VF index, and advanced glaucoma intervention study score. Treatment-related adverse events (AEs) were also compared between groups. Overall, 216 POAG or NTG patients were matched into Match Set 1 (72 patients/group), and 177 NTG-only patients in Match Set 2 (59 patients/group) according to: age (mean: 61, 62 years) and sex (male: 53, 56%). There were no statistically significant between-group differences regarding MD slope (*p* = 0.413, *p* = 0.374 in Match Sets 1 and 2, respectively). There were no significant between-group differences/tendencies regarding secondary endpoints. No AEs were serious, and there were no significant between-group differences regarding reported AEs. In patients with POAG or NTG, long-term tafluprost, travoprost, or latanoprost showed similar effects. All three prostaglandin analogs had good long-term safety profiles.

## 1. Introduction

Glaucoma is a progressive optic neuropathy that causes optic nerve head and visual field (VF) loss. Elevated intraocular pressure (IOP) is considered the principal and only modifiable risk factor for glaucoma, and it is often given a significant role during treatment decisions [1]. Besides IOP, many factors such as age, myopia, metabolic syndrome, etc. are known to involved as risk factors in the pathogenesis of glaucoma [2]. Given that hazards from exposure or accumulation of the effects of risk factors increase over time, the incidence of primary open-angle glaucoma (POAG) is expected to increase continually as life expectancy extends globally. In Korea and Japan, normal-tension glaucoma (NTG; IOP ≤ 21 mmHg) is the most commonly reported type of glaucoma [3,4]. It was reported that the NTG prevalence was 77% among OAG patients in Korea in the Namil Study, and the NTG prevalence was 92% in Japanese patients in the Tajimi Study [4]. In NTG patients, glaucoma damage occurs despite IOP being within the normal range, and it is reported that the progression of glaucoma can be further delayed with additional IOP lowering [5].

Prostaglandin analogs (PGAs) have been shown to be the most popular first-line treatment [6]. Moreover, the role of latanoprost in VF preservation has been recognized [7]. Indeed, this UKGTS trial showed that incident progression was reduced by 41% with the use of latanoprost [7]. A recent study reported that the most widely prescribed treatment of choice for NTG in Korea was PGA monotherapy [8,9]. Commercially available PGAs are tafluprost, travoprost, and latanoprost. There are known variances in clinical efficacy among these PGAs [10], and there is currently a lack of comparative clinical evidence between various PGA products in the real world regarding their effectiveness and tolerability, especially with long-term use.

This study explores the comparative real-world effectiveness of long-term treatment with widely used PGA products—tafluprost, travoprost, or latanoprost—on IOP reduction and various indices for monitoring the status of glaucomatous VF progression in NTG patients using data collected from standard automated perimetry throughout 10 tertiary hospitals in Korea.

## 2. Materials and Methods

### 2.1. Ethics and Consent

The study protocol was approved by the Institutional Review Board (IRB) committee of each study center, following the principles of the Declaration of Helsinki. The requirement to obtain patients’ informed consent was waived, based on the premise that data confidentiality was well maintained and patient privacy was adequately protected.

### 2.2. Study Design and Patients

**Figure 1:**
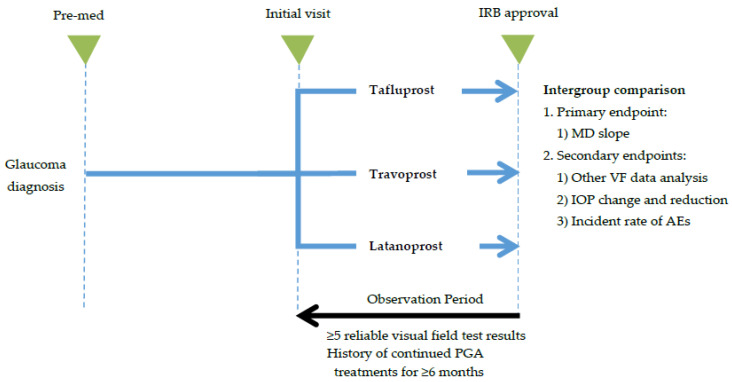
Study flow.

This was a retrospective, multicenter (10 sites in South Korea) cohort study to investigate and compare the long-term effect of three PGA ophthalmic solutions: 0.0015% tafluprost, 0.004% travoprost, and 0.005% latanoprost, in patients with POAG or NTG (Figure 1). Data were collected retrospectively from electronic medical records (EMRs) for patients with POAG or NTG who received PGA ophthalmic solutions as primary monotherapy as initial treatment between January 2010 and June 2016. Study participants were included sequentially from the date of IRB study approval for chart review. Match Set 1 was selected by matching age and sex based on the lowest number of control groups in the full analysis set (FAS) in patients with POAG or NTG. Similarly, Match Set 2 was selected by matching the FAS in patients with NTG. The method of selecting the match set was the SAS macro, percentage match (optimum algorithm) method of Bergstralh and Kosanke [11].

Inclusion criteria included: (1) aged 19 years or older, (2) diagnosis of POAG or NTG, (3) history of administering one of the three above-mentioned PGA ophthalmic solutions to be used in this study before study initiation and existence of at least five reliable VF data points measured with Humphrey perimeter. Exclusion criteria included: (1) history of filtration or any other ocular surgery, (2) presence of pathologies that may affect VF test results, (3) additional administration of medications that may affect IOP during the study period, such as corticosteroids, calcium channel blockers, angiotensin-2 receptor blocker, dorzolamide, or brimonidine, (4) dosage change of concomitant treatments that have been received and could substantially impact IOP or VF.

Additionally, the NTG diagnosis for selection of Match Set 2 was based on the following criteria: (1) typical glaucomatous optic neuropathy with corresponding VF loss; (2) open anterior chamber angles; (3) normal untreated IOP level (≤21 mmHg). Glaucomatous optic neuropathy was determined based on characteristic optic disc and/or retinal nerve fiber layer (RNFL) changes, such as the presence of diffuse or localized rim thinning, rim notching, and an RNFL defect. Typical glaucomatous VF defects, which were assessed by a Humphrey automated perimetry (Swedish Interactive Threshold Algorithm [SITA], standard 24-2/30-2 program) on two occasions, were made based on Anderson’s criteria: (1) the Glaucoma Hemifield Test (GHT) is “outside normal limits”, (2) three contiguous non-edge points on the pattern deviation plot within the Bjerrum’s area with *p* < 0.05, one of which is *p* < 0.01, and (3) pattern standard deviation (PSD) with *p* < 0.05 [12]. Glaucoma severity was divided into early, moderate, and severe according to mean deviation (MD) value (early; >−6 decibel (dB); moderate −6~−12 dB, severe; <−12 dB) [13].

### 2.3. Statistical Analysis

The analysis of efficacy was performed on Match Sets 1 and 2 with last observation carried forward (LOCF). The analysis of safety was performed on the safety set (all subjects enrolled in this study).

The primary objective of the study was to evaluate the rates of VF progression via MD slope expressed in dB/year. Secondary objectives were changes in other VF parameters, including MD, IOP, PSD, VF index (VFI), and the advanced glaucoma intervention study (AGIS) score from baseline. MD, PSD, VFI, and AGIS score [14] were evaluated using the parameters from Humphrey perimetry analyzer. In addition, all adverse events (AEs) reported in the EMRs were recorded for safety and tolerability evaluation.

In this study, a propensity matching approach was used to balance two major confounding factors between the groups: age and sex. Baseline characteristics were reported in mean ± SD or number of patients (%). Comparisons of continuous and categorical variables between the three treatment groups were performed by analysis of variance (ANOVA) and Chi-square tests, respectively. The first time that functional deterioration was found was regarded as the endpoint in survival analyses. *p* values less than 0.05 were considered statistically significant. All statistical analyses were conducted using SAS software (SAS Institute, Cary, NC, USA) V9.4.

## 3. Results

**Figure 2:**
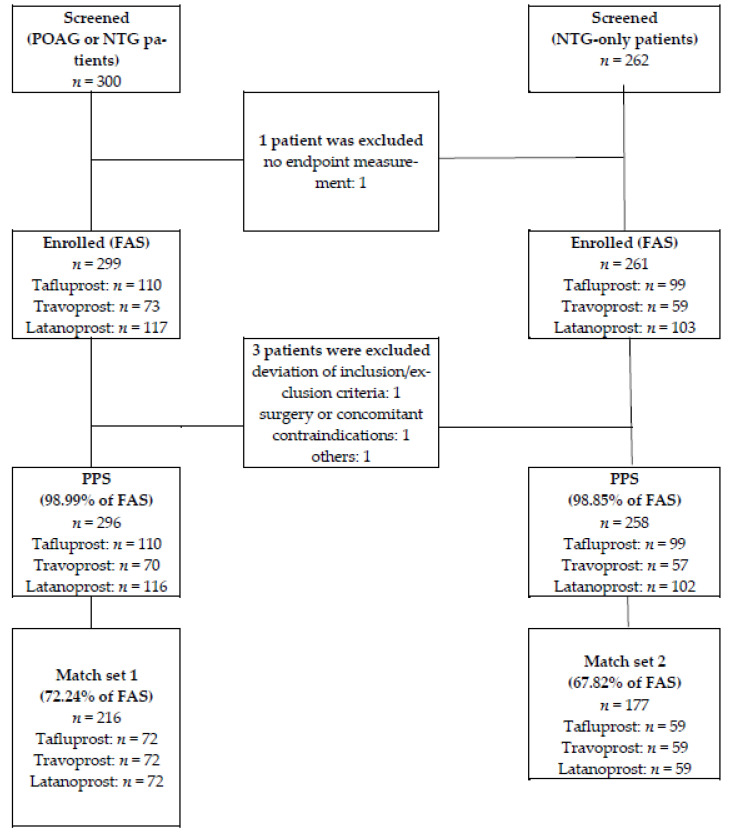
Disposition of POAG or NTG patients (Match Set 1) or NTG-only patients (Match Set 2).

A review of EMRs identified 299 eligible patients (tafluprost, *n* = 110; travoprost; *n* = 73; latanoprost, *n* = 117), who started their first treatment for POAG or NTG between January 2010 and June 2016. Of these, adjustment for age and sex differences between groups using the propensity matching approach resulted in 216 (72.24% of the FAS) patients were included in Match Set 1 (Figure 2). A total of 262 NTG patients were enrolled in the NTG group, and 261 patients were included in the FAS. Of these, 177 (67.82% of the FAS) patients were included in Match Set 2 (Figure 2). The follow-up duration of Match 1 was 4.92 ± 0.27 years (3.5~5 years) in the tafluprost group, 4.85 ± 0.42 years (3.0~5 years) in the travoprost group, and 4.98 ± 0.1 years (4.5~5 years) in the latanoprost group. The follow-up duration of Match 2 was 4.92 ± 0.26 years (3.5~5 years), 4.87 ± 0.34 years (3.5~5 years), and 4.97 ± 0.13 years (4.5~5 years) in the tafluprost, travoprost, and latanoprost groups, respectively.

Table 1 compares the demographic and clinical characteristics of the three subgroups (tafluprost, travoprost, and latanoprost groups) in Match Sets 1 and 2. In Match Set 1, 38 (52.78%) of the subjects who participated in this study were men in the three subgroups, respectively. The average age was 61.04 ± 10.64 years in the tafluprost group, 63.24 ± 11.66 years in the travoprost group, and 61.76 ± 11.17 years in the latanoprost group.

In Match Set 2, ages were 60.27 ± 11.04, 61.83 ± 11.98, and 61.39 ± 11.86 years for tafluprost, travoprost, and latanoprost, respectively. Regarding sex, the proportion of males in the tafluprost, travoprost, and latanoprost arms was 55.93%, respectively.

### 3.1. Effectiveness

Mean changes in MD slope and MD, IOP, PSD, VFI, and AGIS score from the initial visit to the last visit are presented in Table 2.

In Match Set 1, the MD slope was 0.04 ± 0.51, −0.07 ± 0.54, 0.02 ± 0.39 dB/year for tafluprost, travoprost, and latanoprost, respectively, and there was no statistically significant difference between the three groups (*p* = 0.413). There were changes (mean ± SD) at the last visit compared to the initial visit; IOP −1.89 ± 2.77, −2.11 ± 2.16, −2.02 ± 2.88 mmHg; VFI −1.46 ± 4.85, −2.53 ± 9.01, −0.46 ± 5.27; AGIS score 0.00 ± 3.45, 0.00 ± 3.39, −0.06 ± 2.00 for tafluprost, travoprost, and latanoprost, respectively. Furthermore, of these, there was no statistically significant difference between the three groups (*p* = 0.898, 0.255, 0.991) (Table 2).

**Figure 3:**
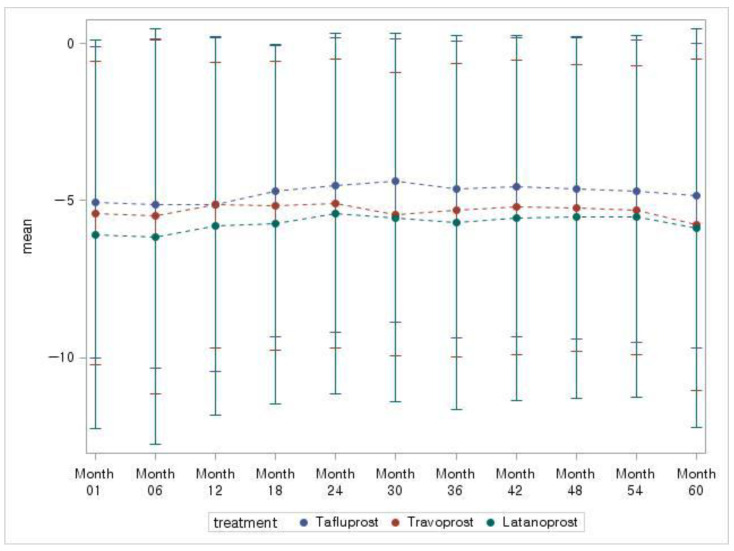
Time course of MD change (Match Set 2).

In Match Set 2, the MD slope was 0.05 ± 0.59, −0.07 ± 0.54, 0.04 ± 0.48 dB/year for tafluprost, travoprost, and latanoprost, respectively. During the study, MD remained relatively stable over time, and no statistically significant difference was observed in any of the three groups (*p* = 0.374) (Figure 3 and Table 2).

**Figure 4:**
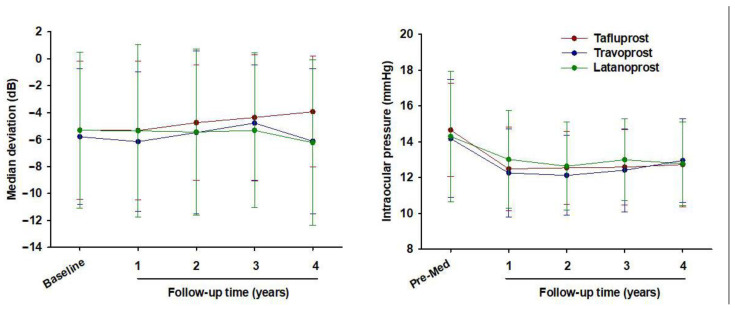
Median deviation (**left**) and intraocular pressure (**right**)–time curves in patients diagnosed with NTG, who received long-term treatment with three PGAs (tafluprost (*n* = 58), travoprost (*n* = 58) or latanoprost (*n* = 58)) and completed the study (Match Set 2).

There were changes (mean ± SD) at the last visit compared to the initial visit: IOP −2.20 ± 2.64, −2.11 ± 2.16, −1.86 ± 2.95 mmHg; VFI −1.14 ± 6.16, −2.53 ± 9.01, 0.23 ± 7.13; AGIS Score −0.17 ± 3.87, 0.00 ± 3.39, −0.36 ± 2.52 for tafluprost, travoprost, and latanoprost, respectively. Furthermore, of these, there were no statistically significant differences between the three groups (*p* = 0.765, 0.171, 0.843) (Table 2). In addition, IOP showed a reduction after 1 year of treatment, and the reduced IOP remained stable until the end of the study (Figure 4).

Overall, there were no significant tendencies between the three groups regarding the change in MD, PSD, and VFI, in Match Sets 1 and 2 (Table 2).

### 3.2. Safety

There were reported AEs; 13 cases from 8/110 patients in the tafluprost group, 20 cases from 9/73 patients in the travoprost group, and 11 cases from 10/117 patients in the latanoprost group (Table 3). The most frequently reported AEs were eye disorders in each group (11, 7, and 8 cases in the tafluprost, travoprost, and latanoprost arms, respectively), and all AEs were not serious. There were no significant between-group differences.

## 4. Discussion

This study demonstrated similar efficacy with respect to mean MD value and MD slope from the baseline and safety among tafluprost, latanoprost, and travoprost groups over the follow-up period. Although many clinical studies have compared the efficacy and safety of two PGAs among the three, these studies also did not show consistent significant differences between each of the PGAs in reducing IOP or progression of VF defects [15,16,17,18].

Previous clinical studies with relatively short-term follow-up have reported possible variances in terms of IOP-lowering effect among different PGA products. Uusitalo et al. reported that, after changing from latanoprost to a tafluprost formulation, tafluprost exhibited a similar IOP-lowering effect and better tolerance [19]. Recently, Faseeh et al. reported in POAG subjects that latanoprost, travoprost, and tafluprost show similar efficacy in terms of reducing mean IOP and diurnal IOP fluctuation in subjects with POAG [20]. IOP is the most important factor associated with the progression of glaucoma. PGAs reduce IOP through a mechanism that makes the extracellular matrix reconstruction to increase the uveoscleral outflow facility of aqueous humor. PGA activates prostaglandin F2a, matrix metalloproteins that are located in ciliary muscle and decompose collagens, resulting in dilation of ciliary muscle, which lowers the resistance of uveoscleral outflow and increases the outflow facility of aqueous humor, resulting in reduced IOP [21,22]. In our study with long-term follow-up, no statistically significant differences were observed between the three treatment groups in terms of IOP changes at the end of the follow-up period. Some studies corroborate the fact that IOP still remains a major risk factor for structural or functional change. Yoshikawa et al. suggested that mean IOP reduction is a key modifiable factor to mitigate the risk of VF progression in NTG patients [23]. In the CNTGS study [24], which has provided some of the most prominent evidence to support the need for robust IOP reduction in NTG patients, it was reported that a 30% reduction in IOP resulted in a significantly decreased risk for VF progression in glaucoma patients, regardless of low baseline IOP. In a study of NTG patients receiving topical medical treatments [25], patients with a higher mean IOP reduction from baseline (i.e., percentage IOP reduction > 22.1%) experienced less progression than patients who achieved a lower mean IOP reduction (i.e., percentage IOP reduction < 13.3%). Wang and Singh suggested lowering IOP further as the most effective way of slowing the progression of glaucoma in patients with low IOP [26]. In the present study, the mean IOP reduction from baseline in NTG patients for the three PGAs, tafluprost, travoprost, latanoprost, was −2.20, −2.11, −1.86 mmHg, respectively, which translates to a rate of IOP reduction of 14.03%, 14.70%, and 12.28%, respectively. Although the rate of IOP reduction achieved in our study is lower than that observed in the above studies, the corresponding rate of VF progression among medically-treated glaucoma patients in our study did not appear to be remarkable over a five-year period of follow-up. It is thought that one of the reasons for the low level of IOP reduction was that our subjects had low baseline IOP compared with previous studies. Considering that glaucoma is caused by the stress of IOP that is higher than the IOP threshold that the optic nerve and lamina cribrosa can tolerate, absolutely low IOP can certainly be seen as a factor that prevented glaucoma progression. Given that glaucoma did not progress if the IOP was <17 mmHg in POAG subjects [27], further studies about safe IOP in low-teen NTG are needed.

Determining VF progression, which is the only functional test in glaucoma, is important and related with the functional endpoint of new treatments for clinical trials and the key to effective clinical disease management [28]. Monitoring the VF status of patients is conducted after testing over a period of time. However, considering the variability of the VF test, appropriate analysis of VF progression measurements requires a suitable observation period and number of follow-up tests to achieve statistical power [29]. In the Early Management Glaucoma Trial and Collaborative NTG study, the mean rate of progression was −1.08 dB/year and −0.2 to −2 dB/year, respectively [23,30]. In a study by Heijl et al., among glaucoma patients under clinical care, the mean rate of progression was −0.80 dB/year [31]. The current study shows a lower degree of VF progression rate than previous reports. None of the three groups appeared to exhibit a significant rate of MD deterioration during the follow-up time in the current long-term study, and the difference between the three groups did not reach statistical significance. This finding may reflect limitations related to the follow-up duration, relatively small sample size, and a relatively higher proportion of NTG patients included in the current study. It is also thought that the fact that they were subjects who had been using the same drugs for a considerable period of time without any problems may have also accounted for the findings. Another reason may be that subjects in our study were relatively younger aged patients with early-stage glaucoma. Since there were as an insufficient proportion of advanced-stage cases in our study, subgroup evaluation by glaucoma stage was not feasible. In addition, the inclusion of subjects with early-stage glaucoma who did not necessarily require treatment cannot be excluded. According to the EMGT report, glaucoma severity (worse MD) is a risk factor for glaucoma progression [30]. Furthermore, in the UKGTS study, only 34% of participants in the untreated group showed VF progression during two years of follow-up [7]. In our study, one reason for the low progression rate may be that most subjects included had early-stage glaucoma. However, the EMGT report showed that the pressure reduction decreased the risk by half. In addition, some studies showed that a significant number of NTG patients did not progress even without treatment [32,33]. The initiation of treatment in patients with early glaucoma may be controversial due to possible overtreatment. The distinction between patients who will not progress without treatment and patients who must be treated is an area where further research is needed.

PGAs are known to be the most efficacious drugs for reducing IOP, while this is associated with higher rates of adverse reactions such as conjunctival hyperemia or deep sulcus [34,35]. Of course, in our study, there were some cases with hyperpigmentation and long cilia. However, there were no serious AEs which resulted in the need to stop instillation of eyedrop. This may reflect the fact that our enrolled subjects had been using the same drugs for a long time without serious complications.

The current study has several limitations. Basically, enrolled subjects in this study had been using the same drugs for a long time without any serious problems. This design might have had an effect on the results. It is known that both the frequency of changing medication and poor medication adherence are glaucoma progression risk factors and, hence, may have confounded the results [27,36]. Fajgenbaum and Ansari reported that PGAs were the most popular first-line treatment in 73% of patients [6]. The mean duration of follow-up was 56 months (24–180 months), and only 27% of patients required only first-line therapy, and 52% of patients required at least third-line therapy during the course of their follow-up. Regarding safety parameters, all of the reported AEs, including patient complaints from the EMRs, were collected for the current study. However, since the information was collected retrospectively, some of the non-serious AEs, such as frequency of conjunctival hyperemia and dry eye, were not clearly evaluated or may have been missed. This can be considered one of the limitations of retrospective chart review studies. However, the rather low frequency of reported AEs can also be interpreted as the relatively infrequent occurrence of severe AEs that led to treatment discontinuation. Thus, we have given more consideration to the effect on IOP lowering and VF progression than to tolerability aspects. Long-term and large-scale prospective studies are needed to overcome this point. Most of the subjects included had NTG; therefore, our results may not be applicable for individuals with high-tension glaucoma. Due to the long follow-up and retrospective design, unmanaged confounders such as self-medication, lifestyle, social-economic status, could have influenced the results [37]. Additionally, patients who adhered to the study the drug due to the positive drug response could have been selected for the analysis. Considering the extensive period required for the current analysis, retrospective design was selected as a rational approach from a practical point of view.

In conclusion, this multicenter, retrospective, cohort study demonstrated that, although there are some nuanced differences, all three PGAs seem to allow robust and comparable IOP reduction as well as no statistically significant differences on the indices related to the rate of VF progression in Korean glaucoma patients over a long-term follow-up period during which all three PGAs showed sufficient VF preservation function alone. This supports the postulate that the three PGA treatments are sufficient as a first-line treatment in Koreans. Therefore, a future prospective clinical trial that compares the benefits of switching to another PGA versus continuing the same PGA after a treatment period of approximately three years or more, may be valuable.

## Figures and Tables

**Table 1:** Clinical characteristics of POAG or NTG patients (Match Set 1) and NTG patients only (Match Set 2).

	Match Set 1			Match Set 2		
	Tafluprost (*n* = 72)	Travoprost (*n* = 72)	Latanoprost (*n* = 72)	Tafluprost (*n* = 59)	Travoprost (*n* = 59)	Latanoprost (*n* = 59)
**Age (years)**	61.04 ± 10.64	63.24 ± 11.66	61.76 ± 11.17	60.27 ± 11.04	61.83 ± 11.98	61.39 ± 11.86
**Gender, *n* (%)**						
Female	34 (47.22)	34 (47.22)	34 (47.22)	26 (44.07)	26 (44.07)	26 (44.07)
**Glaucoma Type, *n* (%)**						
NTG (IOP < 21 mmHg)	65 (90.28)	59 (81.94)	63 (87.50)			
**Glaucoma Stage, *n* (%)**						
Early (MD > −6 dB)	53 (73.61)	48 (66.67)	51 (70.83)			
Moderate	12 (16.67)	17 (23.61)	7 (9.72)			
Advanced (MD < −12 dB)	7 (9.72)	7 (9.72)	14 (19.44)			
**MD at initial visit (dB)**	−5.02 ± 4.81	−5.44 ± 4.64	−5.50 ± 5.80	−5.06 ± 4.95	−5.41 ± 4.82	−6.08 ± 6.19
**Baseline IOP (mmHg)**	15.18 ± 3.28	14.67 ± 2.83	15.54 ± 3.96	15.18 ± 3.30	14.49 ± 2.74	14.98 ± 3.67

Note: Data presented as mean ± SD. Abbreviations: IOP: intraocular pressure; MD: mean deviation; NTG: normal-tension glaucoma; POAG: primary open-angle glaucoma; SD: standard deviation.

**Table 2:** Comparisons of endpoint change between the three PGAs in patients with POAG or NTG patients (Match Set 1) and NTG patients only (Match Set 2). All data are presented as mean ± standard deviation.

	Match Set 1				Match Set 2			
	Tafluprost (*n* = 72)	Travoprost (*n* = 72)	Latanoprost (*n* = 72)	*p*-Value ^†^	Tafluprost (*n* = 59)	Travoprost (*n* = 59)	Latanoprost (*n* = 59)	*p*-Value ^†^
**IOP (mmHg)**								
Initial visit	15.18 ± 3.30	14.49 ± 2.74	14.98 ± 3.67	-	15.69 ± 3.11	14.49 ± 2.74	14.74 ± 3.53	-
Last visit	13.29 ± 1.78	12.36 ± 2.07	12.97 ± 2.67	-	13.49 ± 1.96	12.36 ± 2.07	12.93 ± 2.67	-
Change	−1.89 ± 2.77	−2.11 ± 2.16	−2.02 ± 2.88	0.898	−2.20 ± 2.64	−2.11 ± 2.16	−1.86 ± 2.95	0.765
**MD (dB)**								
Initial visit	−4.63 ± 4.40	−5.41 ± 4.82	−5.66 ± 5.94	-	−5.06 ± 4.95	−5.41 ± 4.82	−6.08 ± 6.19	-
Last visit	−4.50 ± 4.83	−5.78 ± 5.28	−5.58 ± 6.18	-	−4.85 ± 4.85	−5.78 ± 5.28	−5.88 ± 6.35	-
Change	0.14 ± 2.48	−0.38 ± 2.63	0.08 ± 1.96	0.432	0.21 ± 2.86	−0.38 ± 2.63	0.20 ± 2.40	0.390
**MD Slope (dB/year)**	0.04 ± 0.51	−0.07 ± 0.54	0.02 ± 0.39	0.413	0.05 ± 0.59	−0.07 ± 0.54	0.04 ± 0.48	0.374
**PSD (dB)**								
Initial visit	5.67 ± 4.01	6.25 ± 4.06	6.53 ± 4.40	-	5.96 ± 4.17	6.25 ± 4.06	6.68 ± 4.36	-
Last visit	5.92 ± 4.06	6.35 ± 4.18	6.54 ± 4.24	-	6.36 ± 4.17	6.35 ± 4.18	6.44 ± 4.21	-
Change	0.26 ± 2.13	0.10 ± 1.90	0.00 ± 1.67	0.751	0.40 ± 2.18	0.10 ± 1.90	−0.24 ± 1.85	0.215
**VFI**								
Initial visit	87.74 ± 12.73	87.00 ± 13.95	87.60 ± 14.06	-	87.60 ± 13.88	87.00 ± 13.95	86.43 ± 14.78	-
Last visit	88.45 ± 12.37	84.69 ± 16.28	85.11 ± 17.53	-	87.55 ± 12.69	84.69 ± 16.28	84.54 ± 18.20	-
Change	−1.46 ± 4.85	−2.53 ± 9.01	−0.46 ± 5.27	0.255	−1.14 ± 6.16	−2.53 ± 9.01	0.23 ± 7.13	0.171
**AGIS Score**								
Initial visit	3.72 ± 4.53	5.08 ± 5.64	4.70 ± 5.36	-	4.05 ± 4.87	5.08 ± 5.64	5.19 ± 5.22	-
Last visit	3.72 ± 4.50	5.08 ± 5.67	4.63 ± 5.66	-	3.88 ± 4.51	5.08 ± 5.67	4.83 ± 5.40	-
Change	0.00 ± 3.45	0.00 ± 3.39	−0.06 ± 2.00	0.991	−0.17 ± 3.87	0.00 ± 3.39	−0.36 ± 2.52	0.843

^†^ ANOVA *p*-value. MD (median deviation) slope. Data are presented as mean ± SD unless stated otherwise. AGIS: Advanced Glaucoma Intervention Study (worsening is defined by at least 2 units of AGIS score or by at least 2 decibels of MD); IOP: intraocular pressure; MD: mean deviation; NTG: normal-tension glaucoma; PGA: prostaglandin analog; POAG: primary open-angle glaucoma; PSD: pattern standard deviation; SD: standard deviation; VFI: visual field index.

**Table 3:** Ocular adverse events in patients receiving long-term treatment with tafluprost, travoprost, or latanoprost (safety set).

Preferred Term	Tafluprost	Travoprost	Latanoprost
Abnormal sensation in eye	0	1	0
Cataract	0	0	2
Cerebral arteriosclerosis	0	1	0
Chalazion	1	1	0
Chest pain	0	1	0
Chronic gastritis	0	1	0
Ciliary hyperaemia	2	1	0
Conjunctival hyperaemia	2	0	0
Conjunctival irritation	1	0	0
Diabetes mellitus	0	1	0
Diarrhoea	0	1	0
Dry eye	2	0	0
Dyslipidaemia	0	1	0
Dyspepsia	0	1	0
Eye pain	0	1	1
Eye pruritus	1	0	1
Gastritis	0	1	0
Gastritis erosive	1	0	0
Haemorrhagic erosive gastritis	0	1	0
Headache	1	0	1
Helicobacter gastritis	0	1	0
Hypertension	0	0	1
Hyperuricaemia	0	1	0
Ichthyosis	0	0	1
Lacrimation increased	0	0	1
Macular degeneration	0	1	0
Ocular discomfort	1	0	0
Optic disc haemorrhage	0	1	1
Prurigo	0	1	0
Visual impairment	1	1	2
Vomiting	0	1	0

## Data Availability

Restrictions apply to the availability of the dataset used in this study as data were collected retrospectively from electronic medical records (EMRs) of participating study sites; hence, they are available from the corresponding author upon reasonable request. The data are not available publicly due to privacy and ethical restrictions.
